# Molecular Mechanisms of Zika Virus Entry into Host Cells

**DOI:** 10.3390/v18090991

**Published:** 2026-09-09

**Authors:** Haolong Cong, Xin Zhao, Wenhui Li, Rong Lei, Wenjun Zhao, Xiaodong Han

**Affiliations:** 1 Chinese Academy of Quality and Inspection & Testing, Beijing 100000, China; 2 Center for Biosafety, Chinese Academy of Inspection and Quarantine, Sanya 572000, China; 3 College of Life Sciences, Inner Mongolia Agriculture University, Hohhot 010018, China

**Keywords:** Zika virus, E protein, receptor, membrane fusion, entry inhibitor

## Abstract

The World Health Organization classifies mosquito-borne viruses as a major global public health threat with pandemic risk and urges all nations to strengthen pandemic preparedness. As a representative mosquito-borne flavivirus, ZIKV bears prominent pandemic capacity. Its distinctive neurotropic property and capability of vertical transmission create unique pathogenic hazards and transmission risks. Owing to the absence of approved vaccines and targeted antiviral therapeutics, the prevention and management of ZIKV outbreaks remain extremely challenging. Viral entry into host cells marks the very first step of productive ZIKV infection, and mechanistic investigations into this entry process lay an essential theoretical foundation for developing small-molecule agents that block ZIKV cellular entry. This review summarizes recent advances concerning ZIKV host receptor usage, membrane fusion cascades, and entry-targeted inhibitor development; systematically discusses existing inconsistencies and core controversies in cell type-dependent entry pathways, functionally redundant host receptor usage, especially the disputed AXL receptor, and unresolved molecular details of membrane fusion resulting from diverse experimental models and technical limitations; and proposes targeted future research directions to resolve these bottlenecks and clarify the context-dependent ZIKV entry mechanisms.

## 1. Introduction

ZIKV was first isolated in 1947 during yellow fever surveillance from rhesus monkeys in the Zika Forest, Uganda. Its primary transmission vector is Aedes mosquitoes [1]. The natural reservoir of ZIKV has not been fully clarified; accumulating evidence suggests the virus circulates predominantly between wild non-human primates and Aedes mosquito populations [2]. In May 2015, Brazil reported its first locally acquired ZIKV cases, after which the virus rapidly disseminated to dozens of countries across the Americas [3]. Later that year, the World Health Organization (WHO) designated the ZIKV epidemic a Public Health Emergency of International Concern (PHEIC) [4]. The incubation window of ZIKV infection has not been precisely delineated but is generally accepted to range from 3 to 12 days. Roughly 80% of infected individuals remain asymptomatic, whereas only 20% develop mild clinical manifestations [5]. Beyond mosquito-borne transmission, ZIKV can spread via blood transfusion, sexual intercourse, and vertical mother-to-fetus transmission, greatly complicating disease prevention and control strategies [6,7].

ZIKV infection is linked to devastating neurological complications, most notably Guillain–Barré syndrome [8]. The virus penetrates the blood–placenta barrier to infect fetal tissues and impairs embryonic growth, which may culminate in stillbirth or neonatal microcephaly [9]. During the large-scale 2015–2017 South American epidemic, thousands of infants born with microcephaly were documented within a single year [10]. ZIKV is also capable of traversing the blood–testis barrier to colonize the male reproductive tract, where it establishes persistent infection. High viral titers retained in semen enable sustained sexual transmission to female partners [7].

The complete molecular cascade governing ZIKV host cell entry remains poorly characterized, representing a key obstacle to dissecting ZIKV pathogenic pathways and developing targeted entry-blocking antivirals. Viral attachment to cell-surface receptors constitutes the rate-limiting early stage of ZIKV entry. The whole entry cascade—from virion docking at the plasma membrane to lipid bilayer fusion—relies on synergistic interactions with an array of host factors [11,12]. Attachment receptors mediate low-affinity primary binding between virions and cell membranes, followed by high-affinity engagement of secondary host receptors. Upon internalization into endosomes with an acidic luminal environment, ZIKV interacts with endosomal host factors and undergoes irreversible structural rearrangements, priming membrane fusion [13]. Multiple host molecules, such as ITGB4 [14], AXL [15], DC-SIGN [12], and TIM-1 [11,16], have been validated to facilitate ZIKV internalization in a cell-type-specific manner. This review consolidates current knowledge regarding the molecular machinery of ZIKV entry and outlines outstanding research hurdles as well as prospective investigative avenues within this field.

## 2. Structure of ZIKV

The ZIKV virion is a small spherical particle with a diameter of approximately 50 nm [17]. Its layered structure consists of an internal RNA core, capsid (C) proteins, and envelope-associated prM and E proteins from the interior to the exterior of the virion [17,18]. During virion maturation in the trans-Golgi network, the host protease furin cleaves the precursor prM protein into the pr peptide and the mature M protein; the pr peptide shields the E protein fusion loop from premature exposure and dissociates upon virion secretion, yielding fully infectious mature particles [19]. ZIKV possesses a single-stranded positive-sense RNA genome [20], which can be directly translated by the host cellular protein synthesis machinery upon viral entry [21].

**Figure 1:**
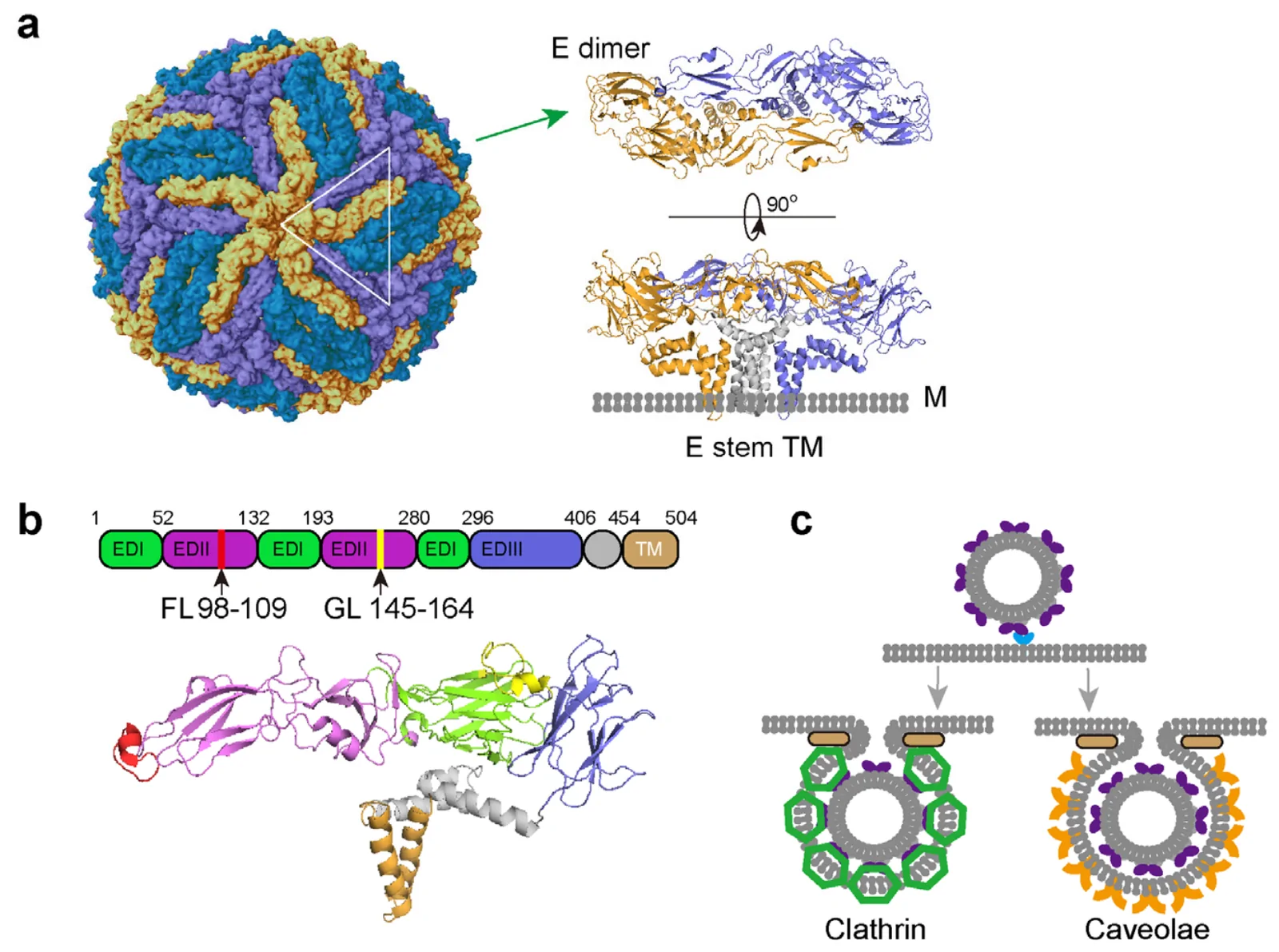
(**a**): Structure of the ZIKV virion, with the E protein homodimers on its surface highlighted in the white box. The E proteins (PDB:5JHM) are arranged in a head-to-tail manner, forming a stable structure. The transmembrane domains of E and M (PDB:5IRE) are embedded in the viral envelope. (**b**): Amino acid positions of the different domains within the E protein. Its EDIII and EDII domains are connected by the EDI domain. The EDII domain contains the fusion loop (red), which becomes exposed in the post-fusion conformation of the E protein for insertion into the host cell membrane. GL 145-164 represents the N-linked glycosylation site (yellow). TM: transmembrane domain (brown). (**c**): ZIKV primarily enters host cells via clathrin-mediated and caveolae-mediated endocytosis. The binding of the virus to the receptor induces the invagination of the plasma membrane, which eventually gets pinched off to form a vesicle. During receptor-mediated endocytosis, endocytic proteins, including the AP2 complex and clathrin triskelion (green) or caveolae (yellow), coat the budding vesicle to form a spherical structure known as a clathrin-coated pit; simultaneously, dynamin (brown) is recruited to the coated pit. Subsequently, dynamin disengages from the pit, leading to the formation of an endosome.

Surface M and E proteins assemble into regular dimeric arrays on the viral envelope and govern viral binding to host cell surface receptors [17,22] (Figure 1a). Under specific microenvironmental conditions, the E protein undergoes substantial conformational rearrangements that trigger fusion between the viral envelope and host cell membrane [23]. As the major viral antigen, the E protein serves as the primary target for host immune recognition, particularly via neutralizing antibodies [24]. Genetic variations in the E protein can markedly alter viral virulence and transmission efficiency [25].

The ZIKV E protein is a type I transmembrane glycoprotein with a molecular weight of approximately 53 kDa [26]. It anchors on the viral surface in the form of head-to-tail homodimers [17], with each virion harboring around 180 E protein copies [17]. These homodimers are arranged in a strict icosahedral symmetry characteristic of mature flavivirus particles, constituting the smooth outer surface of mature ZIKV particles [17]. Structurally, the E protein monomer comprises three distinct functional domains (Figure 1b). Although these domains share high structural homology with those of other flaviviruses, they also exhibit unique ZIKV-specific features [17,27], which are detailed as follows:

EDI (Central domain): As the structural backbone of the E protein, EDI bridges the EDII and EDIII domains and contains a unique N-linked glycosylation site at the Asn154 residue [17]. This glycosylation site is critical for ZIKV neurotropism and vertical transmission [28]. It reportedly mediates viral entry into neural precursor cells and placental cells through specific interactions with host cell surface receptors [28].

EDII (Dimerization domain): EDII is responsible for E protein homodimerization and harbors the conserved fusion loop motif [23]. This elongated finger-like structural terminus contains a stretch of highly conserved hydrophobic amino acids [23] that is essential for viral entry. During infection, the fusion loop inserts into the host endosomal membrane to initiate the membrane fusion cascade [23]. This domain is a major target of flavivirus cross-reactive antibodies [29]. However, due to the high sequence conservation of EDII across flaviviruses, the induced neutralizing antibodies generally exhibit low potency and may even trigger antibody-dependent enhancement (ADE) of infection [29,30].

EDIII (Receptor-binding domain): EDIII is an immunoglobulin-like domain that mediates viral attachment and binding to target host cells [31]. It is the dominant target for the most potent and strain-specific neutralizing antibodies against ZIKV [24,32]. Distinct from the relatively conserved EDII, EDIII displays high sequence variability among different flaviviruses, which defines viral serotype specificity [24]. Antibodies targeting the EDIII domain typically exert robust neutralizing activity with high specificity and a low risk of inducing ADE [24,32].

## 3. ZIKV Entry Pathways

Accumulating experimental observations have reported that ZIKV can utilize distinct endocytic pathways, with the preferred internalization route largely dependent on the specific cell type or cell line examined. The dominant entry pathway utilized by ZIKV is strictly cell-type dependent, which is a major determinant of ZIKV’s broad tissue tropism and diverse pathogenic phenotypes. Notably, certain cell lines such as human glioblastoma T98G cells are capable of supporting dual entry routes, with both clathrin-dependent and caveolae-mediated endocytosis functional for viral uptake [33].

In human microglial (CHME3) and fibroblast (HT1080) cells, ZIKV binds to cell surface receptors and accumulates at clathrin-coated pit sites. The pharmacological inhibitor chlorpromazine blocks ZIKV entry in human glioblastoma T98G cells by suppressing the assembly of cell surface clathrin-coated pits [33]. Similarly, genetic knockdown of the clathrin heavy chain markedly diminishes ZIKV infectivity in both T98G and CHME3 cells [16,33]. In coordination with dynamin and other accessory molecules, clathrin facilitates plasma membrane invagination and membrane scission, resulting in the formation of intracellular endocytic vesicles [33]. These vesicles subsequently mature into early endosomes. Gradual endosomal acidification induces drastic conformational remodeling of the ZIKV E envelope protein [34]. E protein dimers dissociate to expose the hydrophobic fusion peptide within the EDII domain. The exposed fusion peptide inserts into the endosomal membrane, triggering viral-endosomal membrane fusion and the cytoplasmic release of the viral genome to complete viral entry [34,35].

Beyond clathrin-dependent endocytosis, ZIKV utilizes an alternative non-clathrin entry route, caveolae-mediated endocytosis, in T98G cells (Figure 1c) [33]. Multiple experimental approaches have validated this pathway: both caveolin-1-targeted siRNA and overexpression of dominant-negative caveolin-1 efficiently suppress caveolae-mediated endocytosis and substantially impair ZIKV cellular internalization [33,36].

## 4. Receptor Selection of ZIKV

**Figure 2:**
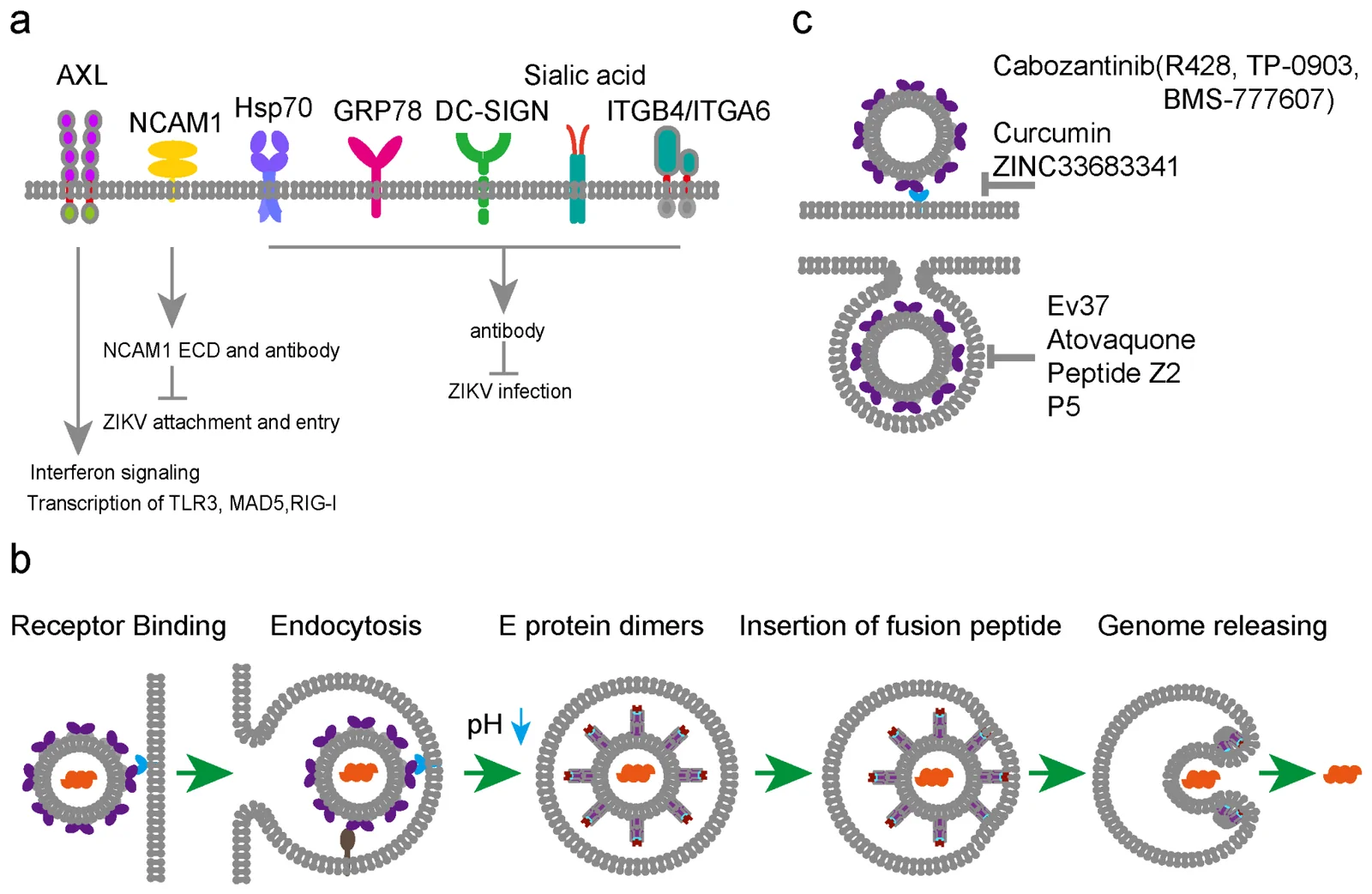
(**a**): ZIKV receptors identified across different cell lines. AXL: astrocytes; endothelial cells; glial cells; neural progenitor cells; microglial cells; Sertoli cells. NCAM1: HEK293T; glioblastoma cells; U-251 MG cells. Hsp70: Huh7.5 cells. GRP78: A549 cells. Sialic acid: Vero cells, hiPSC-derived NPCs, Huh7 cells. DC-SIGN: Vero Cells, SNB-19 Cells. ITGB4: LLC-MK2, Vero cells, A549 cells, Huh7 cells. The ECD of NCAM1 and its corresponding antibodies can inhibit ZIKV adhesion and entry. Antibodies targeting HSP70, GRP78, DC-SIGN, Sialic acid, and ITGB4 have been shown to effectively block ZIKV entry. (**b**): Hypothetical model of ZIKV cell entry based on current studies. ZIKV attaches to receptors (blue) and is internalized via clathrin/caveolae-mediated endocytosis. Endosome acidification triggers E protein (deep violet) conformational changes: dimers reorganize into trimers (lavender), exposing the fusion peptide. This peptide inserts into the endosomal membrane. Further acidification drives trimer refolding into a hairpin structure, forcing viral and endosomal membranes to fuse, thereby releasing the viral genome into the cytoplasm. (**c**): Small-molecule compounds inhibiting ZIKV entry.

Viral entry is orchestrated by the action of multiple host receptor molecules. ZIKV adhesion receptors primarily mediate weak, initial binding interactions between virions and the cell surface, followed by the formation of high-affinity bonds with additional host receptors [34]. Within the acidic endosomal microenvironment, ZIKV undergoes structural rearrangements that render virions fusion-competent. Accumulating evidence has demonstrated that a panel of host molecules, including AXL, NCAM1, HSP70, GRP78, sialic acid, DC-SIGN, and integrins, mediate ZIKV entry in a cell-type-specific manner across diverse human tissues [37,38] (Figure 2a).

TAM Receptor AXL: Expression profiling of neural cells has identified a positive correlation between AXL expression levels and ZIKV neural tropism, supporting the hypothesis that AXL serves as a cellular receptor for ZIKV [39]. Functional cytological assays have further validated that AXL and its ligand Gas6 facilitate ZIKV infection in multiple neural cell types, including glioblastoma cells, human glial cells, and astrocytes [40]. Nevertheless, recent work by Wells et al. demonstrated that robust ZIKV infection persists in neural precursor cells following AXL knockout [41]. Emerging evidence indicates that AXL primarily modulates ZIKV replication by regulating interferon signaling, rather than acting as an indispensable entry receptor [42,43].

AXL indirectly anchors ZIKV via Gas6 bridging and mediates viral internalization through clathrin-dependent endocytosis [44]. The ZIKV–Gas6 complex activates AXL kinase activity, which suppresses host interferon signaling and thereby promotes viral propagation [44,45]. In cutaneous cells, AXL functions as a cell surface attachment factor for ZIKV, and both endocytic uptake and endosomal acidification are required for productive infection [46]. Additionally, AXL enhances autophagosome formation in skin cells, a process that correlates with elevated ZIKV infectivity [11].

In vivo animal studies have further verified the non-essential role of AXL in ZIKV infection. In pregnant mice, ZIKV RNA loads in the brain and spleen exhibited no significant differences among wild-type, Axl-knockout, and Mertk-knockout groups [47,48]. Consistently, non-pregnant Axl-knockout mice treated with IFNAR-blocking antibodies displayed equivalent brain viral levels compared with wild-type controls [48]. Similar viral distribution patterns were observed in the placentas of wild-type, Axl-knockout, and Axl/Mertk double-knockout mice [48]. These findings confirm that AXL and MERTK are not mandatory for ZIKV infection in IFNAR-deficient mouse models. ZIKV can still undergo transplacental transmission and replicate efficiently in placental and fetal tissues in the absence of AXL or MERTK [48].

NCAM1: NCAM1 is abundantly expressed in brain tissues. Ectopic NCAM1 overexpression in HEK293T cells enhances ZIKV attachment and entry, whereas NCAM1 knockdown significantly attenuates ZIKV infection in glioblastoma cells [49]. Co-immunoprecipitation assays have confirmed a direct interaction between NCAM1 and the ZIKV E protein [49]. Both the recombinant NCAM1 extracellular domain and anti-NCAM1 neutralizing antibodies competitively disrupt ZIKV–NCAM binding, thereby markedly inhibiting ZIKV adhesion and internalization in U-251 MG cells [49].

HSP70: HSP70 participates in multiple stages of the ZIKV replication cycle [50]. It is distributed both on the cell surface and in the intracellular compartment [51]. During early infection, cell surface HSP70 interacts with ZIKV virions and promotes viral attachment and entry, thereby enhancing infection efficiency [50,51]. Anti-HSP70 antibodies effectively suppress ZIKV infection and reduce viral progeny production [50,51]. Moreover, incubation of ZIKV with recombinant human HSP70 decreases extracellular viral titers in the supernatant [50,51]. In infected cells, ZIKV double-stranded RNA colocalizes with HSP70, which is integrated into the viral replication complex to support viral proliferation [50,51]. Collectively, HSP70 acts as a multifunctional host factor involved in both ZIKV entry and intracellular replication [50,51].

GRP78: GRP78 is a typical endoplasmic reticulum (ER) lumenal chaperone and can be expressed on the cell surface [52]. A direct interaction between GRP78 and the ZIKV E protein has been identified in human A549 cells [53]. A monoclonal antibody targeting the N-terminus of GRP78 efficiently blocks ZIKV entry, resulting in markedly reduced viral infectivity and replication [54]. Consistently, genetic knockdown of GRP78 impairs ZIKV cellular entry, confirming its critical role in viral cell binding [54]. Intriguingly, GRP78 deficiency disrupts ZIKV-mediated inhibition of host protein translation and alters the subcellular localization of viral replication factories, without affecting viral RNA synthesis [53]. These results suggest that GRP78 serves as a key host regulator during ZIKV infection, primarily by orchestrating the assembly and function of viral replication compartments [53].

Sialic Acid: Cell surface sialic acid is a vital host factor facilitating ZIKV entry [55]. Neuraminidase-mediated removal of surface sialic acid significantly reduces ZIKV infectivity in Vero cells and human iPSC-derived neural precursor cells [55]. Even though sialic acid-deficient Vero cells remain permissive to ZIKV infection, both African (MR766) and Asian (PF13) ZIKV strains exhibit drastically reduced infection efficiency [55]. Similarly, Huh7 cells deficient in 2,3-linked sialic acid display impaired ZIKV entry [55]. Mechanistically, sialic acid is dispensable for initial viral attachment, as comparable ZIKV binding levels were observed in neuraminidase-treated and untreated Vero cells at 4 °C. However, after incubation at 37 °C to allow internalization and pronase digestion to remove uninternalized surface virions, neuraminidase-treated cells showed substantially lower intracellular viral RNA abundance, demonstrating that sialic acid specifically regulates the post-attachment internalization step of ZIKV entry [55].

C-type Lectin Receptor DC-SIGN: DC-SIGN is a canonical C-type lectin receptor that directly mediates ZIKV binding and cellular transmission [56]. It is highly expressed on immature macrophages and dendritic cells [57]. Compared with parental Raji cells and Langerin-expressing Raji cells, DC-SIGN-expressing Raji cells exhibit robust ZIKV binding capacity, which can be specifically blocked by DC-SIGN-targeted antibodies rather than isotype controls [56]. Following co-culture with ZIKV, DC-SIGN-expressing Raji cells, but not Langerin-expressing cells, successfully transmit infectious viruses to recipient target cells within 4 h [56]. Both C-type lectin-blocking polysaccharides and DC-SIGN-specific antibodies efficiently inhibit DC-SIGN-dependent ZIKV infection [56].

Beyond ZIKV, DC-SIGN mediates the cellular entry of multiple flaviviruses, including DENV, WNV, and JEV [56,58,59]. The carbohydrate-recognition domain (CRD) of DC-SIGN binds to the envelope proteins of diverse flaviviruses and is essential for its receptor function [56].

Glycosaminoglycans: Negatively charged cell surface glycosaminoglycans serve as universal adhesion factors to support ZIKV entry [59,60]. Placenta-derived glycosaminoglycans directly interact with the ZIKV E protein and promote viral anchorage to host cells [61]. Notably, glycosaminoglycans mediate cell adhesion for a broad spectrum of viruses beyond flaviviruses, including influenza virus, SARS-CoV-2, and herpes simplex virus, indicating that they act as non-specific, broad-spectrum attachment factors rather than dedicated ZIKV entry receptors.

Integrins: Integrin αvβ5 mediates ZIKV internalization during neural stem cell infection [62]. The cell surface heterodimer integrin α6β4 (ITGA6/ITGB4) binds to the ZIKV E protein and facilitates viral entry in multiple cell types [14]. ITGB4-neutralizing antibodies effectively protect placental tissues from ZIKV infection, reduce embryonic viral titers, and improve embryo survival rates [14]. The ITGA6/ITGB4 complex is widely expressed in central nervous system cells, including glial cells, Schwann cells, and neural stem cells [63,64]. This integrin heterodimer modulates neural stem cell differentiation and strengthens blood–brain barrier integrity by anchoring astrocytes to the vascular basement membrane [65].

## 5. Flavivirus Membrane Fusion Mechanisms

Given the high structural homology between ZIKV and DENV virions, ZIKV is presumed to share a conserved membrane fusion mechanism with DENV (Figure 2b). Following host cell-mediated viral internalization, endosomal acidification acts as the fundamental trigger for flavivirus membrane fusion [66]. The sharp decrease in endosomal pH initiates drastic conformational remodeling of ZIKV E proteins [66]. Protonation of conserved histidine residues destabilizes inter-subunit interactions within E protein homodimers, leading to dimer dissociation into individual monomers. This pH-dependent dissociation is an indispensable prerequisite for unmasking the cryptic fusion loop and freeing individual E protein domains for subsequent large-scale structural rearrangement.

After dimer dissociation, E protein monomers rapidly self-assemble into stable homotrimers, representing a critical transitional intermediate state during membrane fusion [67]. During trimerization, E protein molecules extend outward from the viral envelope, projecting the hydrophobic fusion loop located at the EDII terminus toward the endosomal lipid bilayer [67]. The exposed fusion loops then insert into the host endosomal membrane, anchoring the virion to the endosomal surface. At this stage, the E protein trimer spans two distinct lipid bilayers, with the C-terminal domain embedded in the viral membrane and the fusion loop anchored in the host endosomal membrane, while EDIII remains stably associated with the trimer core.

The most energetically favorable and structurally drastic step of fusion is the jackknife refolding of the EDIII domain. In the extended pre-fusion trimer state, EDIII and the C-terminal stem region undergo a rotational swing of over 100 Å, folding backward toward the viral membrane and central trimer core in a hinge-like manner [68]. This mechanical refolding pulls the virus-tethered host endosomal membrane and the viral membrane into tight juxtaposition. The conformational transition from a high-energy extended intermediate to a thermodynamically stable post-fusion hairpin releases substantial free energy, which overcomes electrostatic repulsion between the two opposing lipid bilayers. The resulting mechanical force and membrane proximity destabilize the outer leaflets of both membranes, driving the formation of a hemifusion stalk. At the hemifusion stage, only the outer lipid leaflets merge, whereas the inner leaflets and luminal aqueous contents remain segregated.

The hemifusion stalk is a transient, unstable intermediate structure. Driven by the formation of low-energy post-fusion E protein hairpin trimers, continuous lipid reorganization ultimately promotes the fusion of inner membrane leaflets and opens a hydrophilic fusion pore. The nascent pore rapidly dilates to release the viral nucleocapsid into the host cytoplasm, completing the entire viral entry process. In the final thermostable post-fusion conformation, E protein trimers adopt a compact hairpin architecture, in which EDIII and the stem region tightly pack against the central EDI–EDII trimer core, locking the fusion process in an irreversible state [69].

## 6. ZIKV Entry Inhibitors

Given that host cell entry constitutes the initial and rate-limiting step of ZIKV infection, targeting E protein-mediated viral entry has emerged as a core strategy for anti-ZIKV therapeutic development. Currently, preclinical ZIKV entry inhibitors are broadly categorized into two classes: neutralizing antibody-based therapeutics that block viral infectivity and small-molecule agents that interfere with viral attachment and downstream entry cascades.

Small-Molecule Compounds Targeting ZIKV Entry: Most reported ZIKV entry inhibitors exert antiviral effects by directly targeting the viral E protein to block structural rearrangement and membrane fusion (Figure 2c). Epigallocatechin gallate (EGCG), a natural polyphenol derived from green tea, exhibits broad-spectrum inhibitory activity against multiple ZIKV strains [70]. EGCG binds specifically to the EDI and EDII domains of the ZIKV E protein, thereby restraining the pH-dependent conformational remodeling of EDIII and abrogating viral entry [71,72]. Nevertheless, the catechol moiety of EGCG may cause off-target cellular inhibition, limiting its specificity. Digitonin, an amphipathic steroidal saponin, suppresses ZIKV infection by disrupting the molecular interaction between the E protein and host cell surface receptors across multiple viral strains [73]. Gossypol, a polyphenolic small molecule, potently neutralizes diverse ZIKV isolates via high-affinity binding to the EDIII domain of the E protein; its derivative ST087010 exhibits improved inhibitory potency while retaining the same targeting mechanism [74]. Atranorin directly targets the ZIKV E protein to impair viral entry and protect human glioblastoma cells from ZIKV-induced infection [75]. Tannic acid binds the E protein and alters key residues responsible for E protein dimerization and membrane fusion, consequently blocking productive ZIKV entry [73].

Curcumin inhibits ZIKV at the early infection stage by binding to the E protein and competitively blocking viral attachment to host surface receptors [76]. It suppresses ZIKV infection in a dose-dependent manner in multiple human cell lines, including HeLa, BHK-21, and Vero cells [76]. Palmatine, a protoberberine alkaloid, also targets the early entry phase by interfering with E protein-receptor binding, viral internalization, and virion stability [73]. Virtual compound screening has identified three small molecules (ZINC23845959, ZINC23400466, and ZINC12415353) that bind the β-octyl glucoside pocket of the E protein and significantly attenuate ZIKV infection in Vero cells [77].

Structure-based drug screening has validated that the small-molecule compound F1065-0358 efficiently inhibits ZIKV replication in Vero cells [78]. All-atom molecular dynamics simulations confirmed its stable binding to conserved residues in EDI (His144, Phe183) and EDIII (Lys301, Tyr305, Asn362), which blocks E protein trimerization and subsequent membrane fusion [73]. This compound exhibits broad inhibitory potential against other flaviviruses [73]. In addition, cyano hydrazone derivatives (3-110-22, 3-110-2, and 3-149-3) target a conserved pocket at the EDI–EDII interface, which is essential for triggering low-pH-induced E protein conformational transitions during viral entry [73].

A competitive amplified luminescent proximity homogeneous assay-based compound library screening identified the small-molecule inhibitor LAS52154459, which impairs E protein-mediated membrane fusion despite relatively moderate antiviral potency [73]. Structure-based computer-aided drug design further discovered a pyrimidine derivative (compound **16a**) that specifically blocks ZIKV internalization and post-internalization processes by interfering with E protein-dependent membrane fusion [73]. Moreover, high-throughput screening of an FDA-approved drug library identified atovaquone as a pan-flavivirus inhibitor capable of suppressing both ZIKV and all four DENV serotypes, highlighting its potential for broad-spectrum flavivirus entry inhibition [73].

Neutralizing Monoclonal Antibodies Against ZIKV Entry: Neutralizing monoclonal antibodies (mAbs) represent promising prophylactic and therapeutic agents for ZIKV infection. A growing panel of ZIKV-specific and flavivirus cross-reactive mAbs has been characterized in recent studies [32]. Structural analyses have revealed that the C10 mAb binds to the E protein intradimer interface, locking the E homodimer structure under acidic conditions and abolishing pH-triggered conformational changes required for membrane fusion [79]. The 2A10G6 mAb targets the conserved E protein fusion loop and confers potent neutralization against ZIKV both in vitro and in mouse models [22]. Five neutralizing mAbs derived from E protein-immunized mice specifically recognize Asian-lineage ZIKV strains by targeting a novel linear epitope within the EDI glycan loop; among these candidates, the 5F8 mAb predominantly blocks early post-attachment entry events [80].

Potent human neutralizing mAbs, including Z23 and Z3L1, have been isolated from ZIKV-infected patients and exhibit robust strain-specific neutralizing activity in vitro [26]. The human mAb ZIKV-117 broadly neutralizes both African and Asian-American ZIKV lineages. In pregnant mouse models, ZIKV-117 treatment markedly reduces placental and fetal viral loads, alleviates tissue damage, and improves host survival [81]. DH1017, a ZIKV-specific IgM mAb isolated from a ZIKV-infected pregnant woman, displays ultrapotent neutralizing activity dependent on its IgM isotype and provides effective protection in mice [82]. In addition, dengue virus patients with prior Japanese encephalitis virus exposure can generate broadly flavivirus-reactive antibodies capable of neutralizing ZIKV [83].

Highly specific ZIKV-neutralizing mAbs (A11 and A42) target the E protein glycan loop and confer robust antiviral protection [84]. The oligomeric state of the ZIKV E protein is critical for eliciting protective humoral immunity; structurally stable recombinant E homodimers induce neutralizing antibodies in mice that recapitulate the epitope recognition patterns of human immune sera from naturally infected individuals [85]. Rational development of ZIKV neutralizing antibodies is largely facilitated by high-resolution structural information of the viral envelope protein [86].

The protective efficacy of multiple human mAbs has been validated in preclinical animal models [87]. For instance, ZIKV-117 treatment significantly ameliorates pathological injury, reduces vertical transmission, and decreases mortality in type I IFN-deficient pregnant mice. Existing studies have also identified a cytotoxic immune signature associated with low post-infection neutralizing antibody titers [88] and characterized germline-derived human mAbs targeting immunodominant ZIKV E protein epitopes [89].

Broadly neutralizing human mAbs with extensive somatic hypermutation can cross-protect against multiple flaviviruses, including ZIKV. Patient-derived ZIKV-specific mAbs target diverse E protein epitopes and exhibit potent neutralizing potency [90]. Longitudinal profiling of human antibody responses during natural ZIKV infection demonstrates that early-induced mAbs predominantly target EDI/EDII with weak neutralizing activity, whereas EDIII-specific neutralizing antibodies gradually dominate the immune repertoire at later infection stages [23].

## 7. Discussion

ZIKV entry into host cells is a key determinant of ZIKV pathogenesis, tissue tropism, and the severe clinical outcomes of ZIKV-associated diseases. Despite substantial progress in characterizing ZIKV infection cycles, the precise mechanisms governing viral receptor engagement, cellular internalization, and membrane fusion remain incompletely elucidated. Notably, the current literature presents considerable discrepancies regarding ZIKV entry pathways, which are largely attributable to the diversity of experimental cellular models and the technical limitations of conventional experimental approaches.

A major unresolved issue in ZIKV entry research is the profound cell-type dependency of viral internalization pathways. Accumulated experimental evidence has demonstrated that ZIKV employs distinct entry routes in different host cell models, leading to inconsistent mechanistic conclusions across published studies. Classical clathrin-mediated endocytosis has been well documented as a dominant entry pathway in human neuronal progenitor cells, renal epithelial cells, and fibroblast cell lines, supporting efficient viral internalization and subsequent productive infection. In contrast, multiple studies have validated caveolae-mediated endocytosis as the primary internalization mechanism for ZIKV in vascular endothelial cells and dermal epithelial cells. Beyond these canonical endocytic routes, alternative entry pathways including macropinocytosis have been identified in placental trophoblasts and immune cells, enabling ZIKV to bypass conventional endocytic machinery and establish infection. Such pathway plasticity is not merely an experimental discrepancy but represents an adaptive viral strategy that facilitates broad tissue tropism, allowing ZIKV to invade diverse somatic and embryonic tissues. Nevertheless, the biological implications of these context-dependent entry mechanisms remain underdiscussed; it remains unclear how distinct entry routes regulate viral replication efficiency, host immune activation, and the development of ZIKV-induced congenital anomalies and tissue-specific pathogenesis.

Beyond the variability of endocytic routes and limitations of detection assays, unresolved controversies over host receptor usage constitute another core bottleneck restricting our comprehensive understanding of ZIKV entry. To date, researchers have identified a panel of disparate attachment factors and co-receptors including AXL, TIM-1, NCAM1, HSP70, GRP78, integrin α6β4, DC-SIGN, sialic acid, and phosphatidylserine receptors, yet no universal, indispensable primary receptor shared across all human tissues has been confirmed. Most candidate receptors exhibit strict cell-type specificity and functional redundancy, which is the root cause of conflicting receptor-related observations across laboratories. A prevailing two-step binding model may reconcile the diverse array of ZIKV attachment factors reported to date: virions first loosely bind low-affinity ubiquitous surface molecules, enabling the virus to “roll” laterally across the cell membrane. This surface scanning continues until ZIKV engages high-affinity functional receptors enriched at clathrin-coated pits, which triggers subsequent productive endocytosis. This rolling mechanism explains why numerous non-exclusive attachment cofactors facilitate viral docking without serving as mandatory entry receptors.

The most representative controversy centers on the TAM receptor AXL. In glial cells, skin keratinocytes, and endothelial cells, AXL acts as a critical attachment cofactor via Gas6 bridging to facilitate clathrin-dependent uptake and suppress antiviral type I interferon responses. However, AXL knockout fails to ablate ZIKV infectivity in neural progenitor cells, cerebral organoids, and placental tissue from both human and mouse models, where phosphatidylserine receptors and integrins compensate for the loss of AXL-mediated binding. Such compensatory receptor networks explain why single-receptor knockout rarely generates complete infection blockade in vitro or in vivo.

Current receptor research also suffers from obvious experimental biases. Most receptor verification experiments are performed in immortalized tumor cell lines with abnormal receptor expression profiles, which cannot fully recapitulate receptor expression patterns in primary human tissues, embryonic organs, or placental barriers. Moreover, the majority of studies adopt single-gene overexpression or knockdown to evaluate receptor function, ignoring the synergistic or competitive crosstalk between multiple host receptors on the same cell surface. Without standardized primary cell and organoid models to systematically compare receptor contributions across tissues, it remains challenging to clarify which receptors dominate ZIKV attachment and entry during natural human infection. Clarifying the hierarchical relationship and tissue-specific distribution of these host receptors will be critical to unraveling the molecular basis of ZIKV tissue tropism and developing receptor-targeted intervention strategies.

Collectively, the discrepancies in reported ZIKV entry mechanisms highlight that conclusions drawn from in vitro studies are highly dependent on the specific cellular experimental system. No single universal entry pathway is conserved across all cell types, and the observed mechanistic differences directly correspond to the distinct expression profiles of cell surface receptors, endocytic machinery, and membrane lipid compositions in different host cells. This model-dependent variability is a key factor underlying inconsistent findings in the field, and researchers must interpret previous mechanistic data with full consideration of experimental model specificity.

Comparing ZIKV and DENV entry mechanisms reveals substantial conserved flaviviral core machinery alongside critical virus-specific distinctions. Both rely on endosomal acidification to trigger E protein dimer dissociation, trimerization and jackknife refolding for membrane fusion, and share broad-spectrum attachment cofactors including DC-SIGN, sialic acid and glycosaminoglycans. Key divergences lie in receptor tropism and pathogenic relevance: unlike DENV, ZIKV uniquely engages AXL, NCAM1 and ITGB4 to traverse placental, blood–brain and blood–testis barriers, driving neurotropism and vertical transmission. DENV entry rarely exploits TAM receptors and lacks the E protein Asn154 glycosylation site central to ZIKV fetal infection. Moreover, DENV entry is less dependent on caveolae-mediated uptake, while ZIKV flexibly switches between clathrin and caveolar endocytosis across cell types. These divergent receptor usages underpin their distinct clinical phenotypes, highlighting that shared fusion machinery cannot fully explain ZIKV’s unique transmission and pathogenic traits.

In addition to experimental methodological caveats, the molecular details governing ZIKV membrane fusion remain incompletely resolved. Additional context on endosomal maturation gradients is also missing: early endosomes maintain a mild pH of 6.0–6.5, while maturing late endosomes and multivesicular bodies (MVBs) reach pH 5.0–5.5, the critical threshold driving E protein dissociation and fusion. Flaviviruses predominantly fuse within late endosomes/MVBs, since early endosomal acidification is insufficient to expose the fusion loop [91]. While endosomal low pH is universally required to trigger fusogenic conformational changes in the ZIKV E glycoprotein, the dynamic kinetics of membrane fusion and potential auxiliary host regulatory factors involved in this process remain poorly characterized. Future investigations should focus on several critical avenues to address the above research gaps. First, it is imperative to systematically compare and reconcile conflicting entry data across diverse cellular models, establishing a cohesive context-dependent model that explains how ZIKV selects specific entry portals in different tissues in vivo. Second, future mechanistic studies should move beyond sole reliance on pharmacological inhibition and integrate multiple orthogonal approaches, including genetic manipulation and high-resolution imaging, to improve the accuracy and reliability of ZIKV entry pathway validation. Third, advanced structural biology approaches, particularly cryo-electron tomography, should be utilized to visualize the in situ dynamics of ZIKV membrane fusion within native cellular environments.

## Data Availability

No new data were created or analyzed in this study.

## Abbreviations

The following abbreviations are used in this manuscript:

ZIKV	Zika virus
WHO	World Health Organization
ADE	Antibody-dependent enhancement
DENV	Dengue Virus
JEV	Japanese Encephalitis Virus
WNV	West Nile Virus
FDA	Food and Drug Administration
